# Solid-State NMR Validation of OPLS4: Structure of PC-Lipid Bilayers and Its Modulation by Dehydration

**DOI:** 10.1021/acs.jpcb.4c04719

**Published:** 2024-12-09

**Authors:** Milla Kurki, Alexey M. Nesterenko, Nicolai E. Alsaker, Tiago M. Ferreira, Sami Kyllönen, Antti Poso, Piia Bartos, Markus S. Miettinen

**Affiliations:** † School of Pharmacy, University of Eastern Finland, 70211 Kuopio, Finland; ‡ Computational Biology Unit, Department of Informatics, University of Bergen, 5008 Bergen, Norway; ¶ Department of Chemistry, University of Bergen, 5007 Bergen, Norway; § Institut für Physik—NMR, Martin-Luther-Universität Halle−Wittenberg, 06099 Halle, Germany

## Abstract

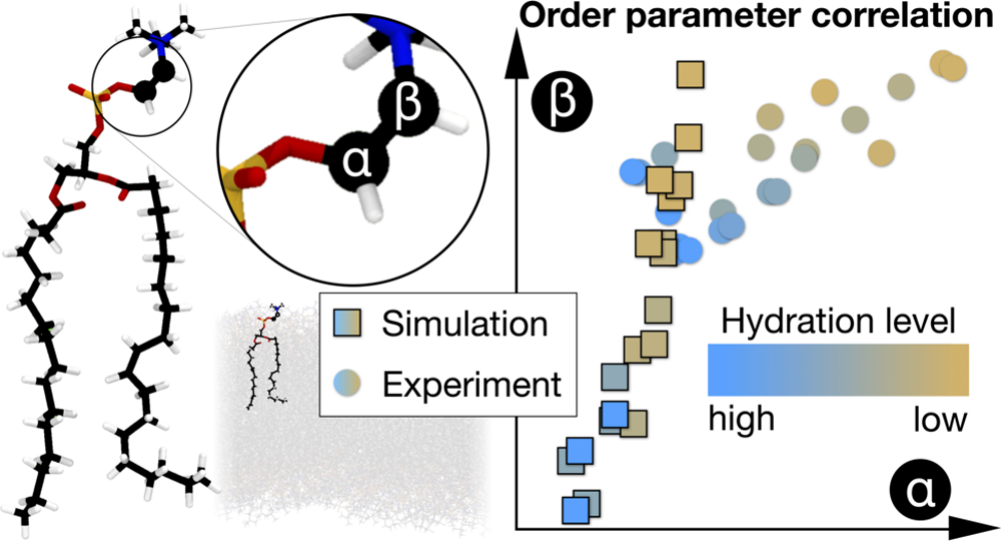

Atomistic molecular dynamics (MD) simulations are a much-used tool for investigating the structure and dynamics of biomembranes with atomic resolution. The validity of the representations obtained is determined by the accuracy and realism of the MD model (force field). Here, we evaluated the proprietary OPLS4 force field of Schrödinger, Inc. against atomic-resolution experimental data, and compared its performance to CHARMM36, one of the best-performing openly available force fields. As a benchmark, we used high-resolution nuclear magnetic resonance (NMR) order parameters for C–H bonds—directly and reliably calculable from MD simulations—measured in phosphatidylcholine (PC) lipid bilayers under varying hydration conditions. Comparisons were made with two dehydration data sets: for saturated (1,2-dimyristoylphosphatidylcholine, DMPC) lipid bilayers from the literature and for unsaturated (1-palmitoyl-2-oleoylphosphatidylcholine, POPC) lipid bilayers measured here. Our findings indicate that OPLS4 reproduces the structure and dehydration response of PC-lipid bilayers fairly well, even slightly outperforming CHARMM36. Both models’ main inaccuracies appear in (1) the order parameter magnitudes in the glycerol backbone and unsaturated carbon segments and (2) the qualitatively differing structural response of the PC headgroup to dehydration compared to experiments. In summary, this work underscores the importance of independent validation for (proprietary) force fields and highlights the striking similarities and nuanced differences between OPLS4 and CHARMM36 in describing biomembranes.

## Introduction

Bilayer lipid membranes are important biological barriers that envelope every cell and most cellular organelles; consequently, their physical properties under various biologically relevant conditions are actively studied using in vitro model lipid systems. Extremely low hydration is a particularly challenging condition that living organisms have managed to overcome: Water content in plant seeds,^1^ as well as in brine shrimp cysts,^2^ can reach just 2% mass weight. While waiting for rehydration, an organism must retain its internal structure, and the integrity of cells and organelles requires integrity of their membranes in the first place. Furthermore, in biological processes that require close membrane alignment, such as membrane fusion, local membrane dehydration occurs even under conditions of water abundance.

Several studies have explored structural changes in dehydrating membranes using, e.g., small-angle X-ray scattering (SAXS)^3^ and solid-state nuclear magnetic resonance with magic angle spinning (MAS ssNMR) experiments,^4−6^ as well as molecular dynamics (MD) simulations.^7−11^ MD simulations are unique among the listed techniques in that they allow visualizing membrane behavior on atomic level: Membranes under biologically relevant conditions are fluids, and possess only short-range molecular order. However, the reliability of MD simulations depends heavily on their verifiability against experiments sensitive to atomic-scale structure. The MD model (force field) used to describe the geometries and energies of molecules needs to veritably depict the structure and dynamics of the biomembrane under the simulated conditions.

One of the most widely used lipid force fields, CHARMM36, was originally parametrized and validated on SAXS measurements of low-hydrated DOPC multilamellar phase,^12^ and was later modified to reproduce better deuterium order parameters of fully hydrated bilayers of six different phospholipids.^13^ Another popular force field, OPLS, with its origins in OPLS-AA and now under development as a proprietary force field by Schrödinger Inc., was parametrized to reproduce solvation of a broad range of small molecules.^14^ As details of the recent OPLS4 lipid parametrization or benchmarks of its performance have not been provided by Schrödinger, we wish to validate it here. To our knowledge, neither force field has been intensively validated for reproducing lipid dehydration, but both are expected to be strong candidates for capturing the molecular details of this crucial biomembrane process.

To assess the quality of force fields we use the C–H bond order parameters *S*_CH_. For lipid bilayers, *S*_CH_ profiles can be experimentally determined from dipolar splittings in ^1^H–^13^C MAS NMR or quadrupolar splittings in ^2^H NMR. Calculating *S*_CH_ from MD trajectories is straightforward, making *S*_CH_ a solid choice for force field performance evaluation:^15^
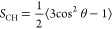
1where the angular brackets denote average over the sampled conformations and θ the angle between the chosen C–H bond vector and the membrane normal. Since *S*_CH_ can be determined for each C–H bond separately, it allows locating limitations of a force field parametrization at exact positions of the lipid structure.

Although lipid dehydration has been studied at least the last 30 years, a complete data set of *S*_CH_ changes upon dehydration has been reported only for DMPC (1,2-dimyristoyl-PC),^4^ a saturated lipid whose melting point is slightly below room temperature. As myristic acid is the shortest-chain fatty acid present (in more than 1%^16^) in the human lecithin composition, DMPC is the most short-chained, and the only saturated, low-melting-point lecithin in the human lipidome. A more standard lecithin for biological studies is POPC (1-palmitoyl-2-oleoyl-PC), which resembles the human plasma membrane in its fluidity and is among the most prevalent membrane lipids in animal cells.^17^ To date, the effect of lowering water content on the POPC glycerol backbone and tail segments has not been reported experimentally—a gap we fill in the present study with ^1^H–^13^C ssNMR experiments that reveal all the *S*_CH_ in POPC.

These novel POPC data, together with the earlier DMPC data, comprise a vast set of high-resolution NMR order parameters, allowing us to evaluate in detail the performance of OPLS4 and CHARMM36 force fields. The experimentally verified simulations then allow us to reveal similarities and differences in the structural responses of the more fluid POPC and the more rigid DMPC membranes to dehydration.

## Methods

### Molecular Dynamics Simulations

We ran 500-ns simulations (1000-ns for OPLS4 POPC at 5 w/l) using the OPLS4^14^ and CHARMM36^13^ force fields with the standard setup for planar bilayers: a tensionless membrane patch in fully periodic boundary conditions. Key simulation details are presented below and in Table 1; further details are available within the simulation files permanently openly available on Zenodo (see IDs listed in Table 1).

**Table 1:** Analyzed Molecular Dynamics Simulation Trajectories^a^

force field	lipid	w/l^b^	*N*_w_^c^	*t*_s_^d^	*t*_a_^e^	open-access IDs^f^^/^g
OPLS3e	POPC	44.3	8859	500	500	805/10183982^18^
		20.0	4000	500	490	815/10183802^18^
		10.0	2000	500	495	816/10183926^18^
		5.0	1000	1000	600	796/10184229^18^
OPLS4	POPC	45.0	9000	500	500	814/10958387
		20.0	4000	500	480	803/10929839
		12.0	2400	500	450	804/10931212
		10.0	2000	500	400	799/10930798
		7.5	1500	500	400	802/10948670
		5.0	1000	1000	650	817/10949612
	DMPC	45.0	9000	500	500	813/11044615
		20.0	4000	500	490	812/11044799
		10.0	2000	500	380	810/11045073
		5.0	1000	500	450	811/11045998
CHARMM36	POPC	44.4	8880	500	460	164/6336691^18^
		20	4000	500	400	819/6335769^18^
		12	2400	500	450	798/10908936
		10	2000	500	425	818/6334005^18^
		7.5	1500	500	290	806/10908508
		5.0	1000	1000	650	795/6333548^18^
	DMPC	50.0	10000	500	500	808/10949663
		20.0	4000	500	480	807/10952391
		10.0	2000	500	450	809/10952764
		5.0	1000	500	350	800/10958132

a All systems had 200 lipids (100 per leaflet).

b Water-to-lipid number ratio.

c Number of water molecules.

d Total simulation time (ns).

e Time used for analysis (ns).

f NMRlipids Databank ID (databank.nmrlipids.fi).

g Zenodo ID (zenodo.org).

Simulations using OPLS4 were run with Desmond in Schrödinger software suite versions 2021.3 or 2022.2.^19,20^ The POPC starting structures were created with the system builder and model system regeneration tools within the software. The standard DMPC starting structures for Desmond were found to be improper due to random chirality; they were recreated on our request by the Schrödinger technical support in March 2024 and fixed for the 2024–2 release. The SPC water model^21^ was used for solvation. Dehydrated systems were derived from the fully hydrated membranes by removing excess water. Default Desmond settings for membrane systems (with 2 fs time step and 10 ps saving frequency) were used, and the presimulation relaxations done using the default membrane relaxation protocol. Temperature was set to 300 K for POPC and 314 K for DMPC. Systems were kept in the NPT ensemble with the semi-isotropic (*xy* coupled separately from *z*) Martyna–Tobias–Klein barostat^22^ and the Nosé–Hoover-chain thermostat.^23^ The default Ewald electrostatics were used. For analysis, the Desmond output files were converted into GROMACS trajectories and topology files with VMD version 1.9.3.^24^

Simulations using CHARMM36 were run with GROMACS versions 2019.5 or 2022.4.^25^ CHARMM–GUI Membrane Builder (charmm-gui.org)^26^ was used to create the systems; the force field parameters were obtained from its outputs. The CHARMM TIP3P water model^27,28^ was used for solvation. Dehydrated systems were derived from the fully hydrated membranes by removing excess water. Simulations were performed with a 2 fs time step and data saved every 10 ps. Temperature was set to 300 K for POPC and 314 K for DMPC. Systems were kept in the NPT ensemble with the semi-isotropic (*xy* coupled separately from *z*) Parrinello–Rahman barostat^29^ and the Nosé–Hoover thermostat.^30,31^ Particle Mesh Ewald^32,33^ (PME) was used for electrostatics.

### Simulation Analysis

From the saved simulation trajectories, C–H bond order parameters *S*_CH_ were calculated with the calcOrderParameters.py Python program (available on the NMRlipids Databank^34^ GitHub repository) that uses the MDAnalysis library;^35,36^ the program calculates *S*_CH_ based on eq 1 for each lipid over time separately, and then the average and the standard error of mean over different lipids. To evaluate equilibration of simulations we used area per lipid (see Figure S4) calculated as the *xy*-area of the simulation box divided by the number of lipids per leaflet.

### Sample Preparation for ssNMR

Required amounts of POPC in chloroform solution (Avanti Polar Lipids, Birmingham, AL, USA) were moved into a weighed Eppendorf tube and freeze-dried overnight to completely remove the solvent. The Eppendorf with the dried lipid was weighed again, the desired amount of deionized water added with Ar(g) bubbling (plus 1–2 μL), and the sample allowed to swell (40°C, 30*′*). Then the sample was mechanically mixed using a thin polished stainless steel rod, the Eppendorf closed with Parafilm to prevent evaporation, and let to equilibrate (40°C, 24 h). The sample then was mixed again and equilibration continued for 48 h, after which samples were packed into Kel-F MAS rotor inserts (Bruker, B4493) by inserting a 100-μL tip into the insert and centrifuging (1000 g, 5*′*). Packed and sealed inserts were stored at 4°C before being measured. For effective packing of the samples in the inserts, we targeted to have approximately 40–50 mg of each sample in total (exceeding the amount needed to fill the whole volume of the insert), taking into account some material loss when transferring the sample from the Eppendorf tube to the insert.

### Solid-State NMR

Solid-state NMR experiments were performed on a wide-bore Bruker Avance III HD 500 MHz spectrometer (Fällanden, Switzerland) equipped with a triple-resonance 4 mm CP-MAS BL4 probe. The recording of experiments was carried out with the Bruker TopSpin 3.6.3 software. Primary processing of spectra was done in TopSpin 4.1.1.

The samples were packed into Kel-F MAS rotor inserts (Bruker, B4493), sealed with plugs and screws, and placed inside 4 mm ZrO_2_ rotors with a Kel-F drive cap (Bruker, H14355). All NMR experiments were performed at 298 K sample temperature, unless otherwise stated, and kept stable within ±0.1 K with a Bruker BCU II–80/60 variable temperature unit (VTU) (Fällanden, Switzerland). The VTU was calibrated using MeOH-d4 (99.8%).^37^

^1^H and ^13^C pulse calibration was done through nutation frequency experiments on the sample of interest spinning at 5 kHz. Adamantane spinning at 10 kHz was used as chemical shift reference for ^1^H (1.85 ppm), and H_3_PO_4_ for ^31^P (0 ppm). Sample hydration was determined by ^1^H MAS (Figure S2). Lipid phase was confirmed to be lamellar by the signal line-shape of static ^31^P spectra.^38,39^

### R-PDLF Experiments

R-type proton-detected local field (R-PDLF) recoupling experiments^40,41^ were recorded at 5 kHz spinning rate, using refocused insensitive nuclei enhancement by polarization transfer (rINEPT)^42^ for polarization transfer and ^1^H broad-band decoupling during acquisition.

The rINEPT delays used were multiples of the MAS period and equal to τ_1_ = 1.2 ms and τ_2_ = 2.0 ms. Rf-pulses were calibrated to give the following nutation frequencies: 45.00 kHz (R18_1_^7^ recoupling pulses), 71.43 kHz (^13^C 90 and 180° pulses), 71.43 kHz (^1^H INEPT and 90°), 35.71 kHz (^1^H broadband decoupling pulses). For each *t*_1_ point, 256 transients were collected over an acquisition time of 50 ms using a 5-s recycle delay. A total of 32 points in the indirect dimension were collected with increments of 18 × 2 × 11.11(1) μs. The R-PDLF spectra were processed as described previously^40^ and the absolute values of *S*_CH_ calculated by dividing the measured dipolar splittings by the ^1^H–^13^C rigid dipolar constant of 22 kHz. The signs of *S*_CH_ values were determined previously by two independent methods for egg yolk PC,^43^ which contains PC molecules with a distribution of distinct unsaturated and saturated acyl chains, and for DMPC:^44^ Both works found the α carbon to be the only segment having positive *S*_CH_.

## Results and Discussion

### Experimental Dehydration Response of POPC Resembles DMPC

We measured all the C–H bond order parameters *S*_CH_ in lamellar-phase POPC–water mixtures at various levels of hydration using ^1^H–^13^C R-PDLF solid-state NMR. Earlier, Dvinskikh et al.^4^ did the same for DMPC using APM-CP (amplitude-and-phase-modulated cross-polarization).

**Figure 1:**
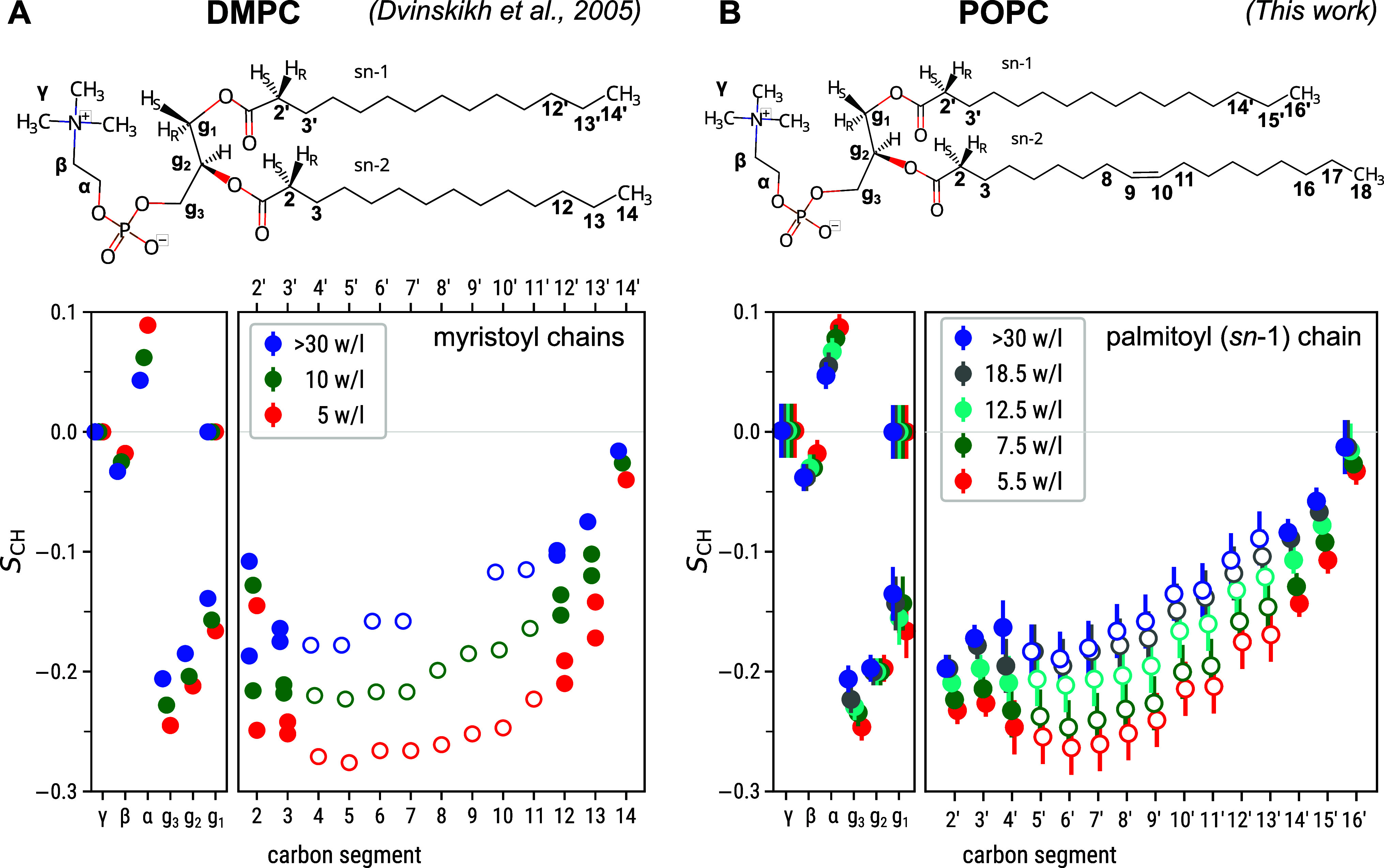
Experimentally observed C–H bond order parameters *S*_CH_ in phosphatidylcholine lipid bilayers at various levels of hydration. (A) DMPC data measured using APM-CP, reproduced from ref (4). Note that in APM-CP, the observed *S*_CH_ are averages over the protons bound to the same carbon; also, *g*_1S_ is independently known to have *S*_CH_ = 0. (B) POPC data measured using R-PDLF in this work; note that the unsaturated oleoyl (*sn*-2) tail data are shown separately in Figure 2. In R-PDLF, protons bound to the same carbon can be observed to fork, i.e., give distinctly differing *S*_CH_. For the *S*_CH_ shown in this figure, we observed forking only for the carbon segment *g*_1_ (but see Figure 2 for the *sn*-2 tail); for other segments, the observed *S*_CH_ of the different protons are equal. Empty symbols denote the *S*_CH_ extracted from the crowded spectral region (see Figure S1 for POPC and Figure 6 of ref (45) for DMPC).

Figure 1 compares the Dvinskikh DMPC data (panel A) to our POPC data (panel B) for the PC headgroup, the glycerol backbone, and the saturated (*sn*-1 in POPC) tails. For all nonzero *S*_CH_, the qualitative response to dehydration is very similar in both lipids: The headgroup *S*_CH_ increase, the backbone and tail *S*_CH_ decrease. An exception is the carbon segment *g*_2_, whose *S*_CH_ appear insensitive to hydration level in POPC, whereas in DMPC the negative *S*_CH_ decreases further upon dehydration.

In quantitative terms, the *S*_CH_ response seen in Figure 1 for POPC is smaller than for DMPC: The median magnitude change from full to lowest hydration is 0.06 over the POPC and 0.08 over the DMPC carbons. The largest quantitative difference between lipid responses manifests in the second-last and third-last saturated-tail carbons, whose mean *S*_CH_ changes roughly 70% more in DMPC than in POPC. In both DMPC and POPC saturated tails, the most pronounced dehydration response occurs midchain.

**Figure 2:**
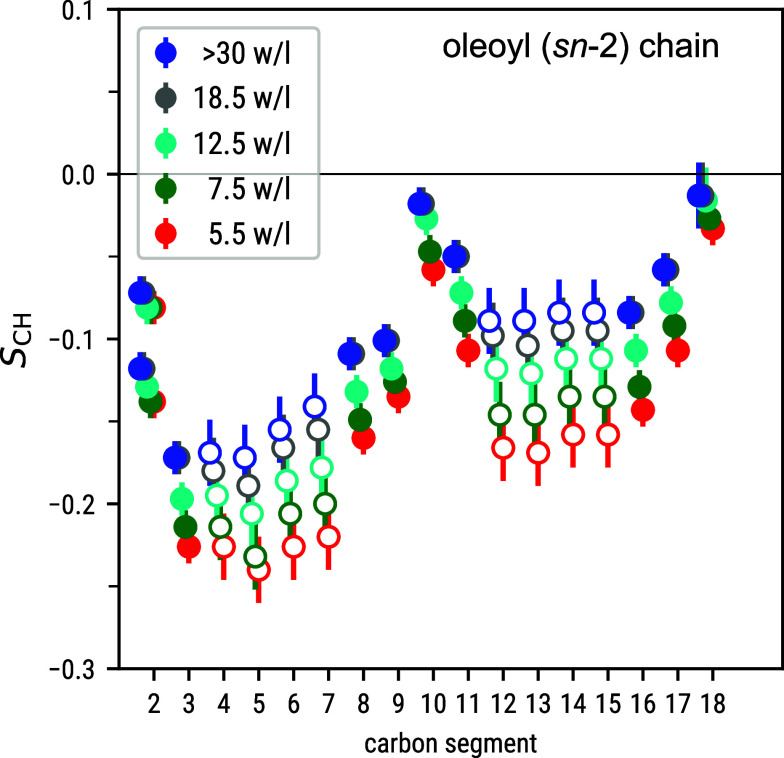
Experimentally observed C–H bond order parameters *S*_CH_ of the unsaturated oleoyl tail in POPC bilayers at various levels of hydration. Note that as the individual carbons in some unsaturated/saturated tail carbon pairs (3/3′, 16/14′, 17/15′, and 18/16′) cannot be distinguished, these values are here identical to those shown in Figure 1B for the saturated palmitoyl (*sn*-1) tail. However, unlike for the saturated tails in Figure 1, here forking on the carbon segment 2 is observed. Empty symbols denote the *S*_CH_ extracted from the crowded spectral region (see Figure S1).

As the midchain carbons reside in a highly crowded part of the ^13^C NMR spectrum (see Figure S1 for POPC, Figure 6 of ref (45) for DMPC), assigning their dipolar splittings (i.e., *S*_CH_) to the corresponding carbons is challenging. Our assignment is based on previous work that used *sn*-1-perdeuterated POPC to separate the *sn*-1 from the *sn*-2 carbons.^40^ Since some intratail ambiguity still remains, Figures 1 and 2 indicate the crowded-region *S*_CH_ with empty symbols.

Figure 2 shows the unsaturated oleoyl (*sn*-2) tail of POPC. Note that whereas the DMPC carbon segments 3/3′, 12/12′, and 13/13′ allowed Dvinskikh et al. to see the two myristoyl tails separately in APM-CP^4^ and R-PDLF,^45^ this was not the case for the corresponding POPC carbon segments in our R-PDLF data: The unsaturated/saturated intertail carbon pairs 3/3′, 16/14′, and 17/15′ cannot be separated (Figure S3) even though the *S*_CH_ can be unambiguously assigned as coming from these pairs. The reason for the lack of resolution in our data is the use of a lower number of points in the indirect dimension in comparison to the experiments performed by Dvinskikh.^4^ The carbon segments 8–11 around the double bond can, however, be clearly identified in the ^13^C spectrum: Their *S*_CH_ have lower magnitudes than the corresponding segments in the saturated tail (compare Figures 2 and 1B), and upon dehydration they change less (mean full-to-lowest-hydration magnitude change 0.05) than the *S*_CH_ in the flanking carbon segments 4–7 and 12–15 (0.08).

Let us next use these high-resolution experimental data to assess the validity of OPLS4.

### Validity of the OPLS4 Force Field

#### Full Hydration

**Figure 3:**
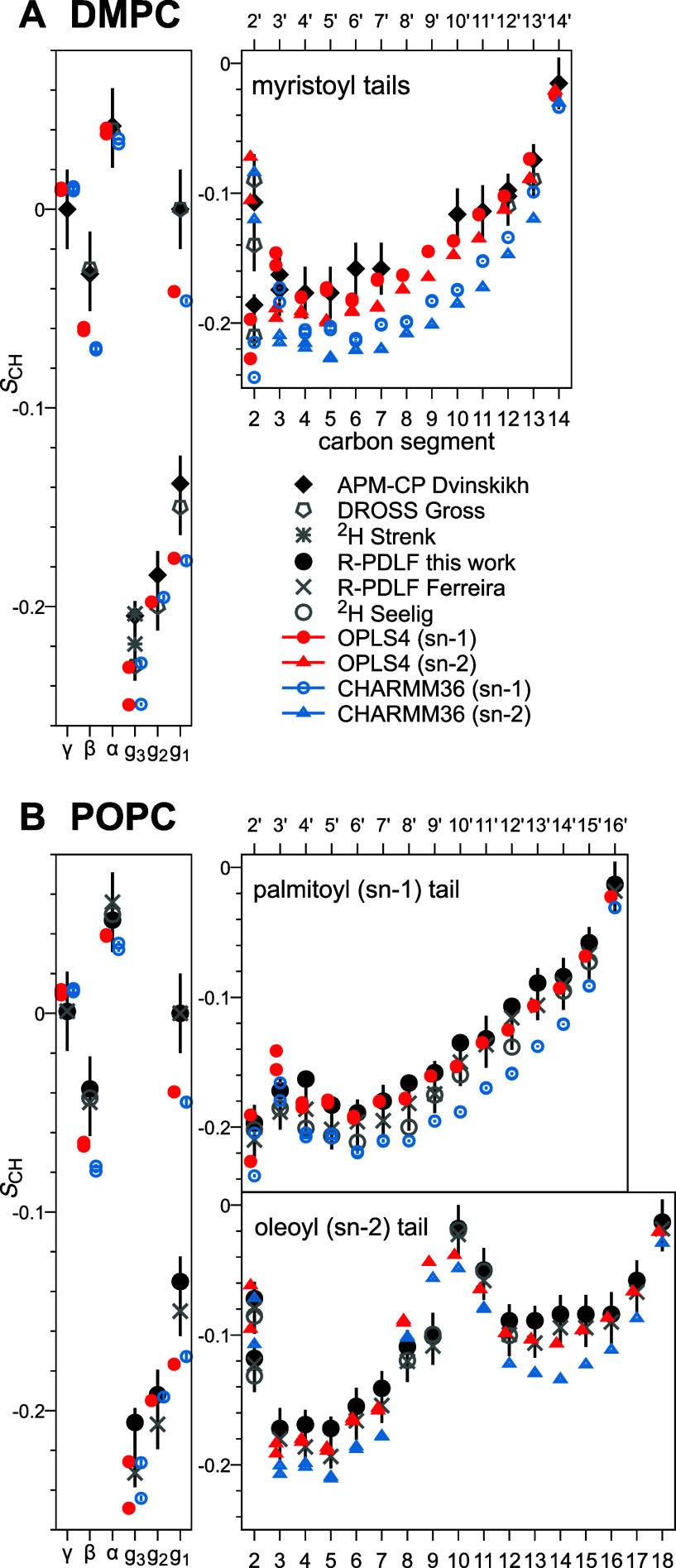
Simulations versus experiments in fully hydrated lipid bilayers of (A) DMPC at 314 K and (B) POPC at 300 K. C–H bond order parameters *S*_CH_ in OPLS4 (red symbols) and CHARMM36 (blue) simulations, as well as in experiments (black/gray) from the literature—DMPC^4,44,47^ (Gross at 303 K, Strenk at 309 K), POPC^40,48,49^ (^2^H NMR of β and α at 296 K)—and from this work. The ±0.02 black error bars indicate where the *S*_CH_ of a well-performing force field should lie.^50^

Figure 3 shows the ability of OPLS4 to reproduce the experimental C–H order parameters *S*_CH_ in fully hydrated DMPC (Figure 3A) and POPC (Figure 3B) lipid bilayers, and contrasts this with the performance of CHARMM36, a state-of-the-art force field for lipid simulations. We see that in the PC-headgroup and glycerol backbone region (left panels) OPLS4 is practically identical to CHARMM36: Just the β carbon segment *S*_CH_ is 0.01 units closer to experiments in OPLS4. In the tail region (right panels) the two force fields are, however, different: Whereas CHARMM36 typically overestimates (as previously reported^18,46^) the *S*_CH_ magnitudes, OPLS4 matches the experimental values, i.e., predicts slightly more disordered acyl chains than CHARMM36.

Despite the very good overall performance, a few mismatches persist in simulations compared to experiments: (i) the *S*_CH_ magnitudes of β, *g*_3_, and especially *g*_1_ carbon segments are overestimated; (ii) the *sn*-1 tail carbon segment 2′ forks too much—as much as the *sn*-2 carbon segment 2, but the latter is also experimentally visible; (iii) the *S*_CH_ magnitude of the unsaturated *sn*-2 tail carbon segment 9 in POPC is underestimated. All of these three mismatches are also observed for CHARMM36.

While keeping these limitations in mind, let us point out a suggestive structural feature seen in both force fields (Figure 3A, see also Figure S5): There appears to be an odd/even variation such that at any even DMPC tail carbon segment (such as 6/6′) the interchain *S*_CH_ difference is smaller than at the neighboring odd segments (5/5′ and 7/7′). This feature cannot be confirmed with our experimental resolution, but it may be related to a previously reported odd/even effect on C–C bond order parameters *S*_CC_.^51^

Let us then turn to the dehydration responses.

#### Dehydration: Headgroup and Backbone

**Figure 4:**
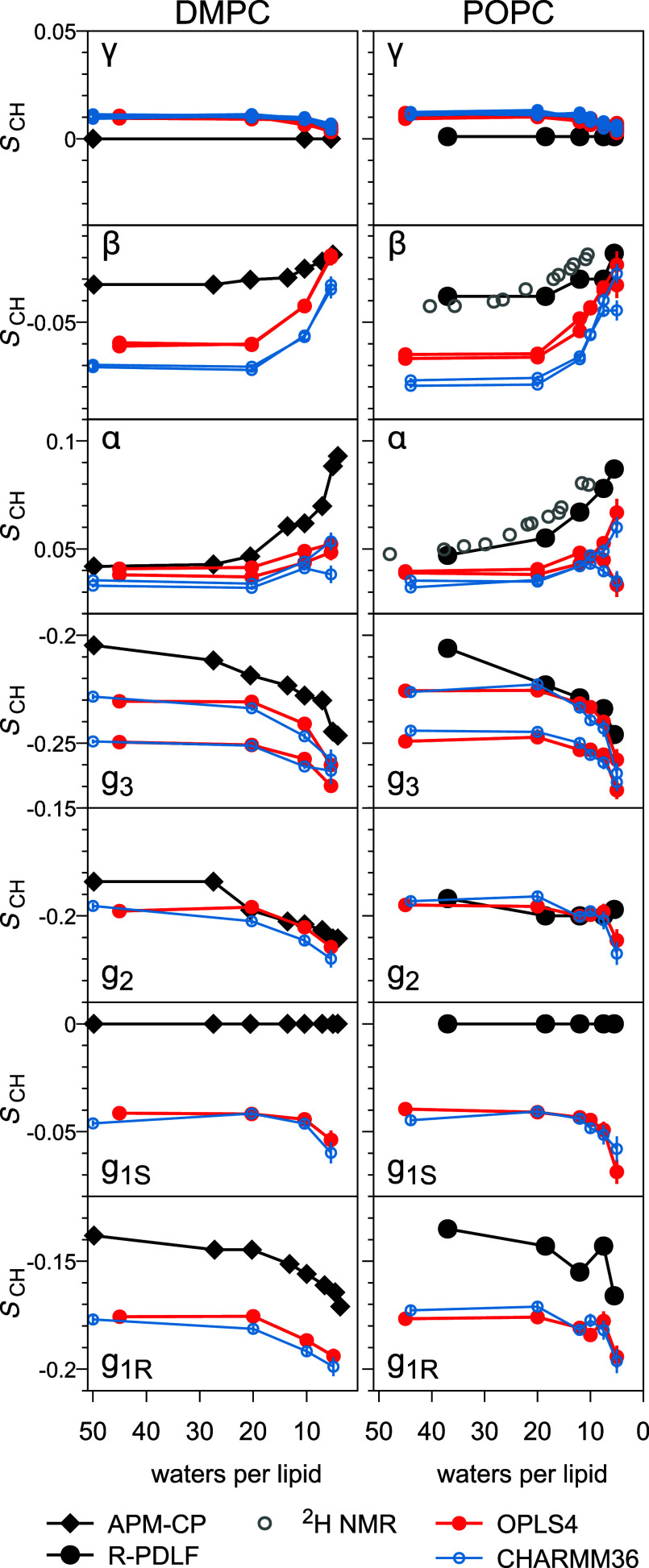
Simulated versus experimental dehydration responses of water-facing carbon segments in DMPC (314 K, left) and POPC (300 K, right) bilayers. C–H bond order parameters *S*_CH_ in simulations (red/blue symbols), as well as in our and literature (DMPC from ref (4) and POPC ^2^H NMR at 296 K from ref (49)) experiments (black/gray).

Figure 4 compares OPLS4 and CHARMM36 in reproducing the experimentally observed *S*_CH_ responses of water-facing carbon segments in PC-lipid bilayers (DMPC left, POPC right) to dehydration. The two force fields behave astonishingly alike. This is rather interesting, especially as the water models were not the same: SPC^21^ and the CHARMM-specific TIP3P.^27,28^ In fact, the most eye-catching difference results from a feature that is inherited from the fully hydrated case—the 0.01 units larger β-carbon *S*_CH_ in OPLS4 (cf. Figure 3).

The only qualitative differences occur at the extreme dehydration of 5 w/l; here *g*_3_ forks only in OPLS4, and DMPC α only in CHARMM36. Few quantitative differences are also observable at 5 w/l: POPC β forks more in CHARMM36, and POPC *g*_1S_ is slightly more negative in OPLS4. But beyond these minutiae, and the perhaps slightly weaker response of OPLS4 in DMPC *g*_2_ and *g*_1R_, the two force fields are—in good and in bad—indistinguishable in their headgroup and backbone responses to dehydration.

Direction of the simulated dehydration response is correct in all water-facing carbon segments—except for the two *S*_CH_ that should remain zero at all hydrations (γ and *g*_1S_, see Figure 4). For these, simulations predict decreases that may fit within the margin of experimental error in γ, but not in *g*_1S_ (even if the dipolar coupling spectrum of the *g*_1_ carbon is less clearly resolved, and thus its *S*_CH_ uncertainty higher, than of the other water-facing carbons).

**Figure 5:**
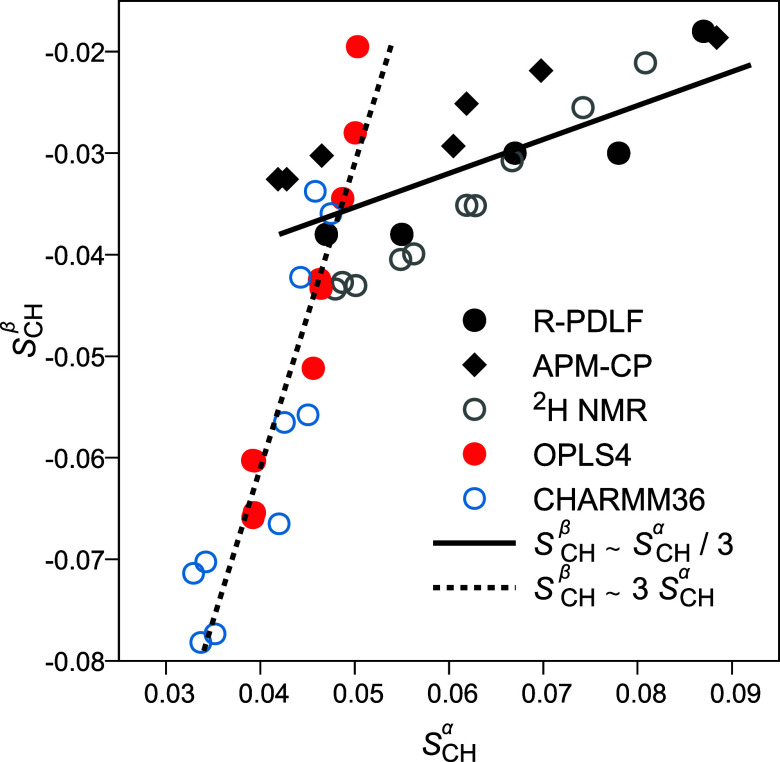
*S*_CH_^β^ versus *S*_CH_^α^ in experiments and simulations. Data include both POPC and DMPC. Experimental data are from this work and refs (4 and 49). Trendlines are not fits.

The (β and α) headgroup *S*_CH_ increase upon dehydration (Figure 4) is thought to result from the headgroup aligning more parallel to the membrane plane,^49^ and is qualitatively reproduced by most MD force fields.^50^ It seems, however, that the mechanism of this structural response differs between simulations and experiments: In experiments β changes less than α, *S*_CH_^β^ ∼ *S*_CH_^α^/3, but in simulations these roles are reversed, *S*_CH_^β^ ∼ 3 *S*_CH_^α^ (Figure 5). Previously such overestimation of the *S*_CH_^β^-versus-*S*_CH_^α^ slope by simulations has been reported for cation binding.^52^ Possible differences in response mechanism are suggested also by the fact that only in simulations the *S*_CH_ tend to fork at the lowest hydrations (particularly visible in the α carbon of POPC) and that experiments show signs of change in headgroup *S*_CH_ already at >20 w/l, but simulations only at <20 w/l.

For glycerol backbone (excluding g_1S_), simulations capture the *S*_CH_ decrease magnitudes rather well (Figure 4). Indeed, the largest quantitative discrepancies manifest not in the responses, but are inherited from the fully hydrated case: The *S*_CH_ magnitudes of *g*_3_, and especially *g*_1_, are too large. (Note, however, that the extents of their full-hydration forkings do agree with ^2^H NMR,^47^ see Figure 3.)

#### Dehydration: Tails

**Figure 6:**
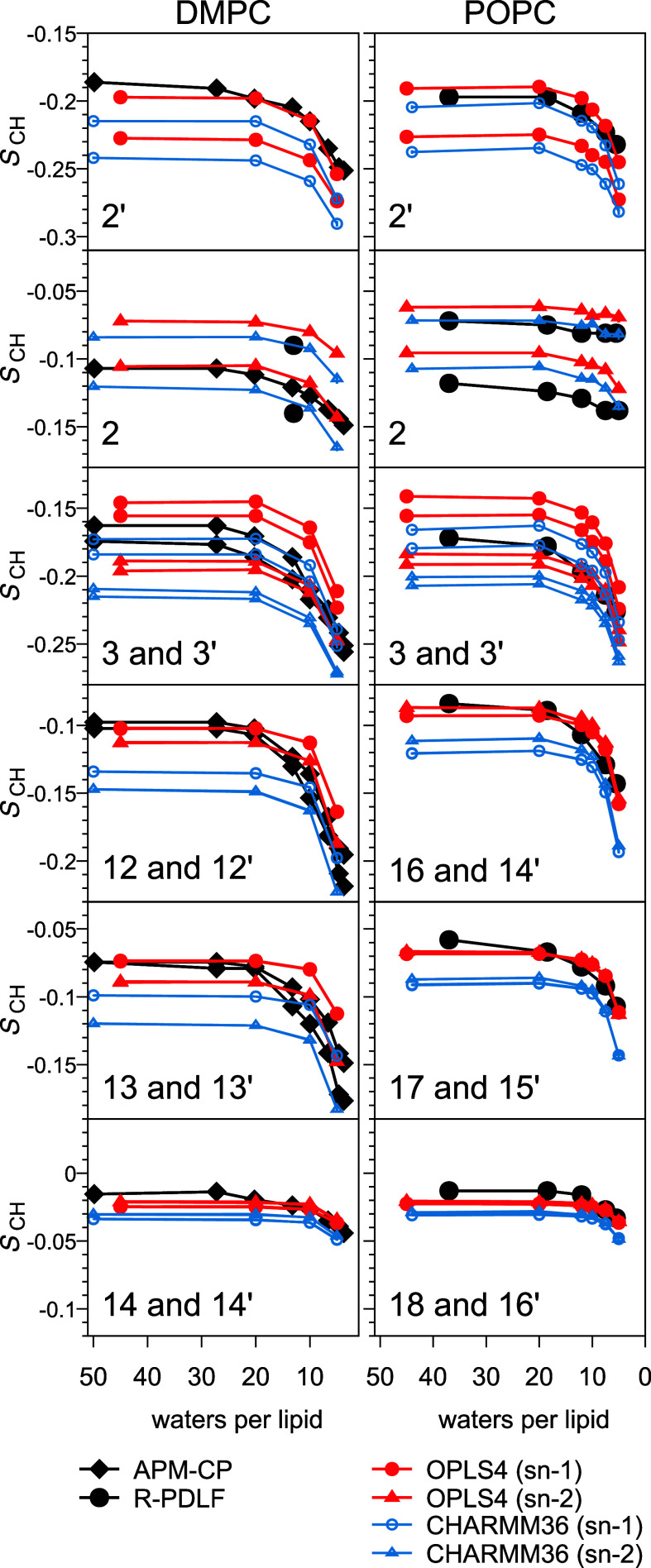
Simulated versus experimental dehydration responses at the base and tip of DMPC (314 K, left) and POPC (300 K, right) lipid tails. C–H bond order parameters *S*_CH_ in simulations (red/blue symbols, each proton’s *S*_CH_ is plotted separately), as well as in our POPC and literature DMPC^4,45^ experiments (black). For the midtail carbon segments, see Figures S6–S8.

Figure 6 displays dehydration effects on *S*_CH_ at the beginning and end of tails in DMPC (left) and POPC (right). The tail responses of the two force fields are seen to be very similar, and their full-to-lowest-hydration *S*_CH_ changes in fair agreement with experiments. However, as the full-hydration *S*_CH_ of OPLS4 match the experiments better (Figure 3), it often also follows the dehydration behavior more closely than CHARMM36. The only clearly opposite behavior to this trend is in the (*sn*-2) carbon segment 2, whose forking both force fields capture well, but CHARMM36 better the magnitude (Figure 6); also in the unsaturated (*sn*-2) segments 8 and 9, whose *S*_CH_ both force fields overestimate at full hydration (but OPLS4 slightly more, see Figure 3), CHARMM36 remains slightly better as dehydration decreases the *S*_CH_ (Figure S7).

Notably, as for water-facing segments (Figure 4), also in tails the *S*_CH_ change only at <20 w/l in simulations, but in experiments already at >20 w/l (Figure 6). This delayed response complicates somewhat the general trend presented by the previous paragraph and—especially when combined with slight deviations from experiments in full-to-lowest-hydration *S*_CH_ magnitude changes seen in all DMPC (underestimation, except in *sn*-2 carbon 2, see Figure S8), some unsaturated POPC (underestimation, Figure S7), and in several saturated POPC (overestimation, Figure S6) tail segments—leads to situations where certain carbon segments become equally well or better described by CHARMM36 at some hydrations. See, e.g., the DMPC carbon segment 13/13′ in Figure 6.

Forking is in simulations observed in four tail carbon segments (2, 2′, 3, and 3′) but experimentally only in segment 2 (Figure 6). For segments 3/3′, the experimental resolution is not sufficient to judge the correctness of the simulational forking. For segment 2′, however, it is clearly too large: Were the simulational value correct, the 2′ forking would be also experimentally observable in R-PDLF—as the equally large forking at segment 2 is, see Figure 6.

The *S*_CH_ difference between *sn*-1 and *sn*-2 tails observed especially in DMPC simulations is clearly overestimated at all hydration levels for 3/3′, and at >20 w/l for 12/12′ and 13/13′ (Figure 6).

As shown (Figures 3 and 6), the lipid tail structures in OPLS4 are closer to experiments than in CHARMM36, whose overestimation of the *S*_CH_ magnitude indicates overly ordered tails, and consequently underestimation of area per lipid. Indeed, in all dehydrated states, CHARMM36 area estimates are lower than OPLS4 by 1–3 Å^2^ (Figure S9, Table S1). Such overshrinking and overestimated tail *S*_CH_ magnitudes in CHARMM36 have been previously reported, e.g., for DPPC (1,2-dipalmitoylphosphatidylcholine) bilayers.^46^ To visualize how the dehydration-induced increase of *S*_CH_ magnitude (Figure 1) and the concomitant decrease in area per lipid (Figure S9) affect the saturated tail geometry, Figure S10 shows histograms of tail end-to-end distance and tail tilt angle at decreasing hydration: As expected, the tails become straighter and orient more along the bilayer normal, but interestingly the distributions change little until <10 w/l, and markedly only at 5 w/l.

#### OPLS4 versus OPLS3e

Upon publishing OPLS4,^14^ there were no reported changes from the older OPLS3e^53^ force field concerning lipid parameters. We found that, at full hydration, the *S*_CH_ in these two force fields are indeed identical (Figure S11), and they do not significantly differ even when high concentrations of salts are added (Figures S14 and S15). At low water levels, however, there seem to be small differences, especially within the headgroup and glycerol backbone (Figure S12). Most noticeably, these lead to the dehydration-induced forking artifact of the α carbon segment (as in CHARMM36, see Figure 4) that did not appear in OPLS3e (Figure S13).

## Conclusions

Atomistic molecular dynamics (MD) simulations are a widely used and highly valuable tool to reason on biomolecular structures and dynamics. Especially among industrial users, the MD engine Desmond by Schrödinger, Inc. has become a popular choice due to its simple user interface. Its proprietary force fields are, however, not widely independently validated; this applies also to its latest all-atom force field, OPLS4. Here, we tested the validity of OPLS4 against atomic-resolution experimental data and contrasted its performance to another industry standard, CHARMM36.

As our benchmark system, we used phosphatidylcholine (PC) lipid bilayers under decreasing hydration, biologically relevant, e.g., in the desiccation and vesicle fusion contexts. Our high-resolution experimental data comprised Nuclear Magnetic Resonance order parameters for C–H bonds, *S*_CH_, which can be directly and reliably calculated from MD simulations. We presented comparisons against a dehydration data set of all POPC lipid bilayer *S*_CH_ measured in this work (see Figures 1B and 2), as well as literature dehydration data sets of all DMPC^4^ and of two POPC^49^ headgroup *S*_CH_.

We find OPLS4 to reproduce the structure of fully hydrated PC-lipid bilayers well, even slightly better than CHARMM36 (Figure 3). This slight superiority further carries over to dehydration responses (Figures 4 and 6). On the whole, the two force fields appear extremely similar—also in their problems. For example, at full hydration they both struggle with the backbone *g*_1_ and unsaturated tail segment *S*_CH_ magnitudes. Further, their PC-headgroup structural response mechanisms to dehydration seem to be similar, but qualitatively different from experiments (Figure 5). For example, the POPC headgroup α carbon response to extreme dehydration has become practically identical in OPLS4 to that in CHARMM36 (Figure 4), although it was more realistic in the older OPLS3e (Figure S13).

To sum up, the pictures the two force fields provide of true PC-lipid bilayers are rather truthful—and almost identical, also in their distortions.

## Data Availability

The scripts for the C–H bond order parameter analysis are freely available at NMRlipids GitHub (github.com/NMRlipids) and the analyzed trajectories are permanently freely available at Zenodo (zenodo.org) with IDs listed in Table 1. Solid-state NMR data are publicly available on the Norwegian Dataverse node (https://doi.org/10.18710/ETWNCU).

## Supporting Information Available

Selected parts of NMR spectra and following MD data: equilibration of areas per lipid, equilibrium areas per lipid, difference of *S*_CH_ between DMPC tails, complete plots of hydrocarbon tail order parameters upon dehydration, distributions of tail lengths and orientations upon dehydration, and comparison of OPLS3e and OPLS4 force fields (at full hydration, at low hydration, and with monovalent and divalent salts). (PDF)
