# Supplementary material for: Solid-State NMR Validation of OPLS4: Structure of PC-Lipid Bilayers and Its Modulation by Dehydration
Source: J Phys Chem B. 2024 Dec 9;128(50):12483–92. doi: 10.1021/acs.jpcb.4c04719 (PMC11664586; doi:10.1021/acs.jpcb.4c04719)
Supplement: Supplementary file 1 — jp4c04719_si_001.pdf [file jp4c04719_si_001.pdf]

Supplementary information: Solid-state-NMR validation of OPLS4: Structure of PC-lipid bilayers and its modulation by dehydration

Milla Kurki,[†,‖] Alexey M. Nesterenko,[‡,¶,‖] Nicolai E. Alsaker,[¶] Tiago M. Ferreira,[§] Sami Kyllönen,[†] Antti Poso,[†] Piia Bartos,[†,⊥] and Markus S. Miettinen[*,‡,⊥]

†*School of Pharmacy, University of Eastern Finland, 70211 Kuopio, Finland*

‡*Computational Biology Unit, Department of Informatics, University of Bergen, 5008 Bergen, Norway*

¶*Department of Chemistry, University of Bergen, 5007 Bergen, Norway*

§*Institut für Physik — NMR, Martin-Luther-Universität Halle–Wittenberg, 06099 Halle, Germany*

‖*M.K. and A.M.N. contributed equally*

⊥*P.B. and M.S.M. contributed equally*

E-mail: markus.miettinen@iki.fi

1 ^{13}C–^{1}H CP/MAS NMR

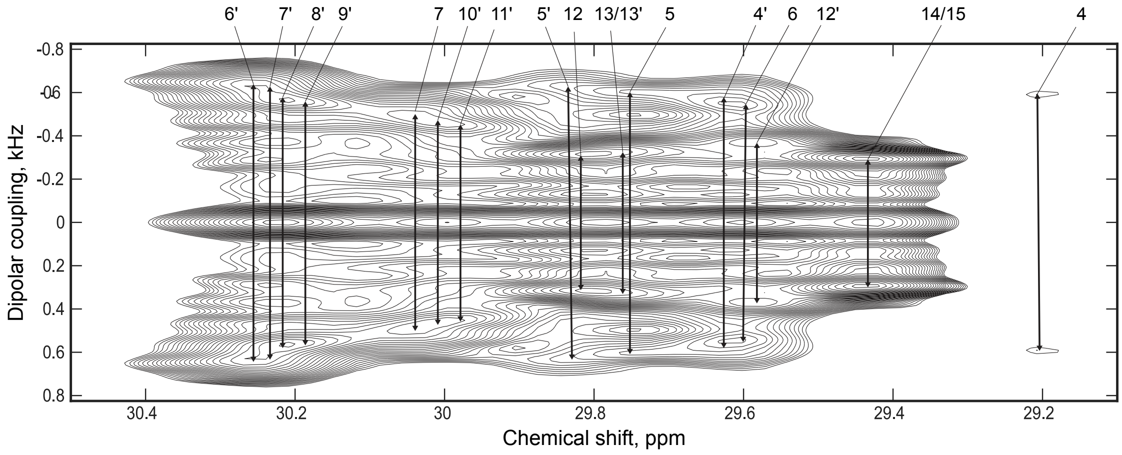

Figure S1: Assignment of 29.1–30.5 ppm region of ^{1}H–^{13}C R-PDLF spectrum, which allows us to determine S_{CH} values for all carbon segments of lipid hydrocarbon chains. Labels are explained at Fig. 1.

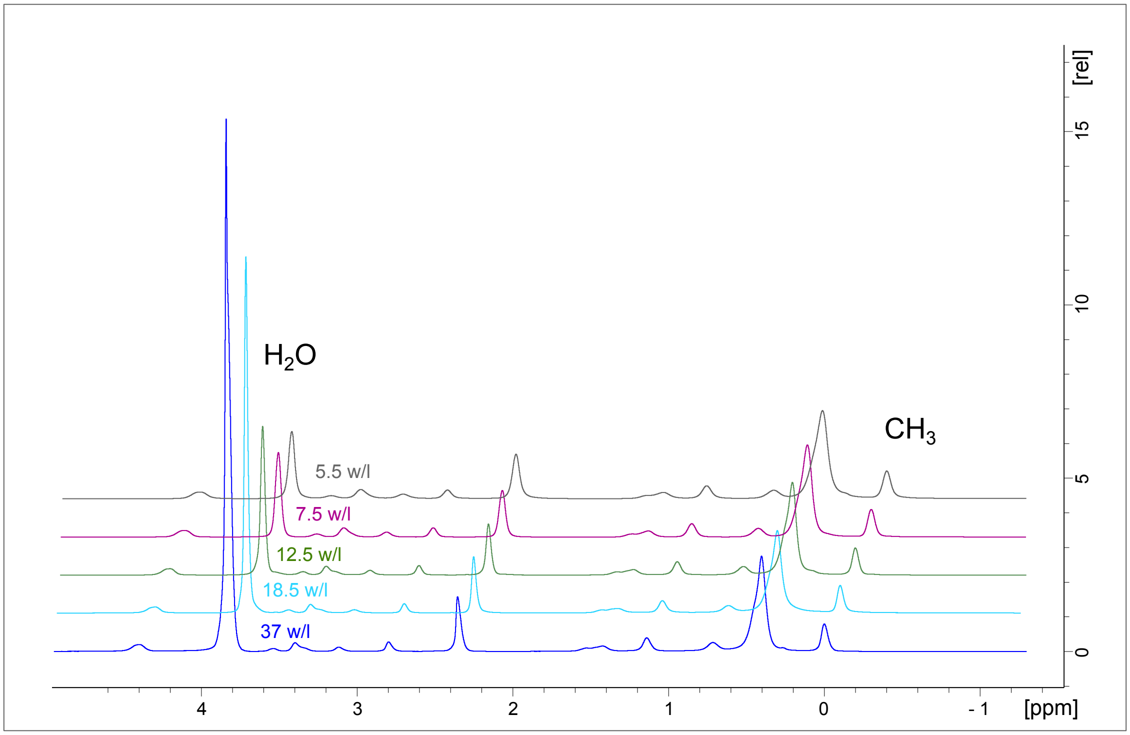

Figure S2: ^{1}H CP/MAS NMR spectrum of all four POPC samples with different hydration states. CH_3 peaks are aligned by intensity.

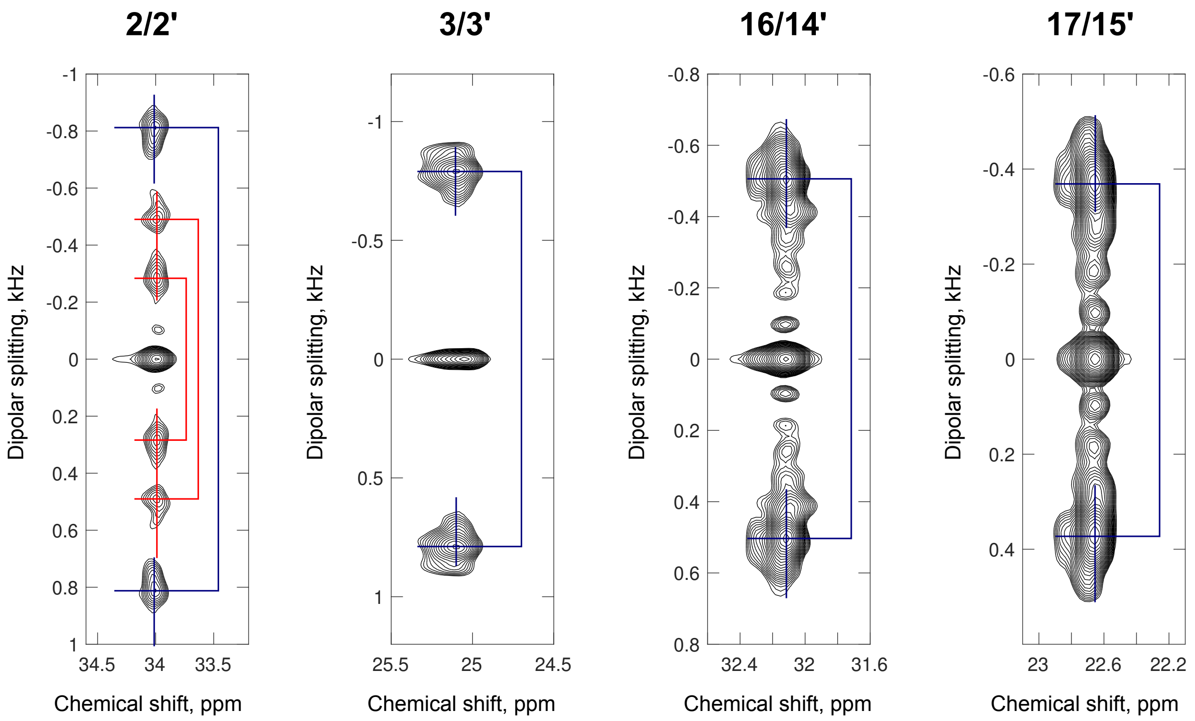

Figure S3: **Dipolar splittings of four pairs of overlayed carbons for the most dehydrated POPC sample** (5.5 waters per lipid). Only for carbons $2/2'$ we clearly see different splittings (red and blue). For the other three pairs, the resolution doesn't allow us to resolve them.

2 System equilibration

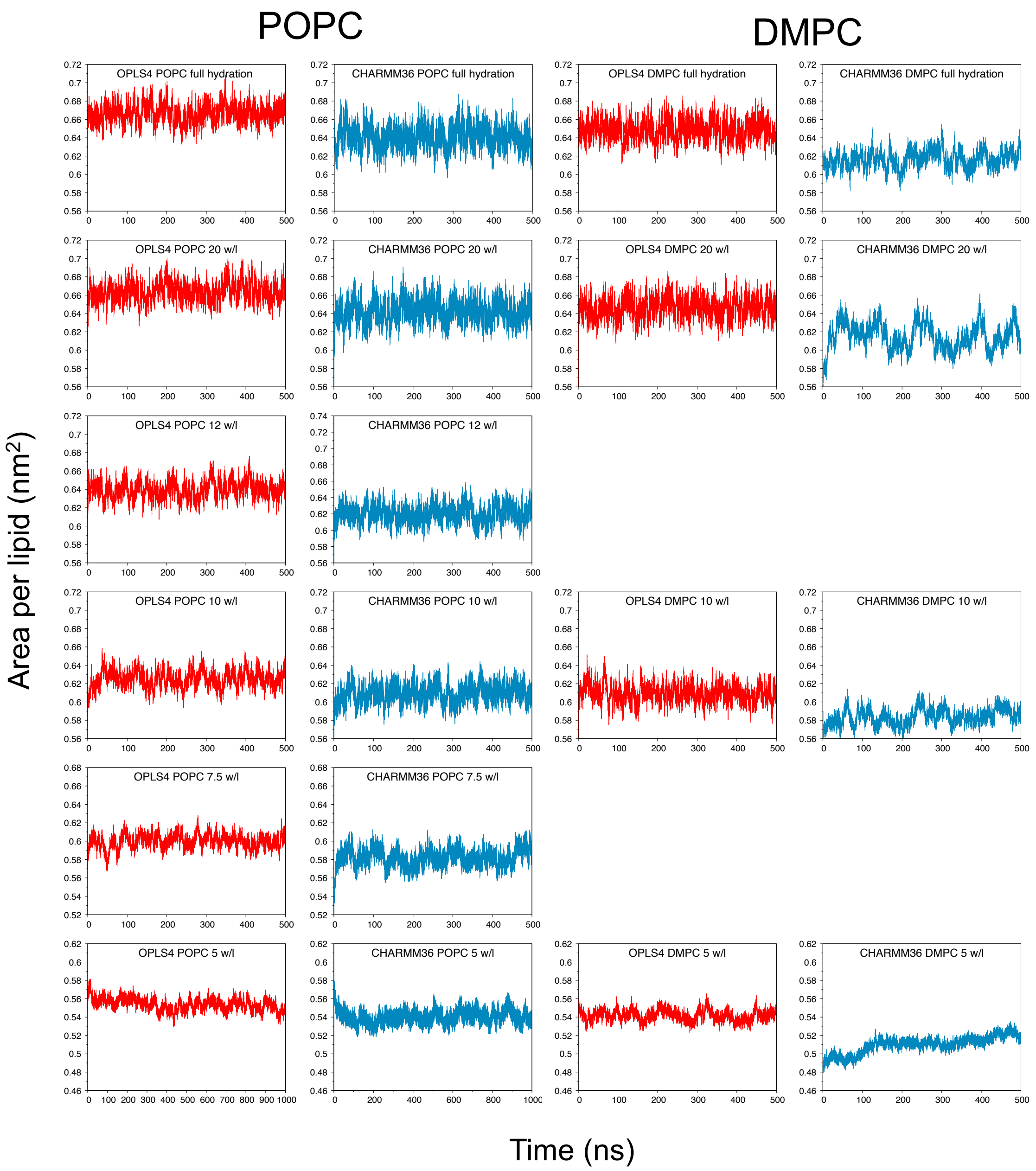

Figure S4: Area per lipid as a function of simulation time in our MD systems. Note that y-axis is different for 5 w/l, 7.5 w/l, and for 10–50 w/l.

3 DMPC interchain difference observed in simulations

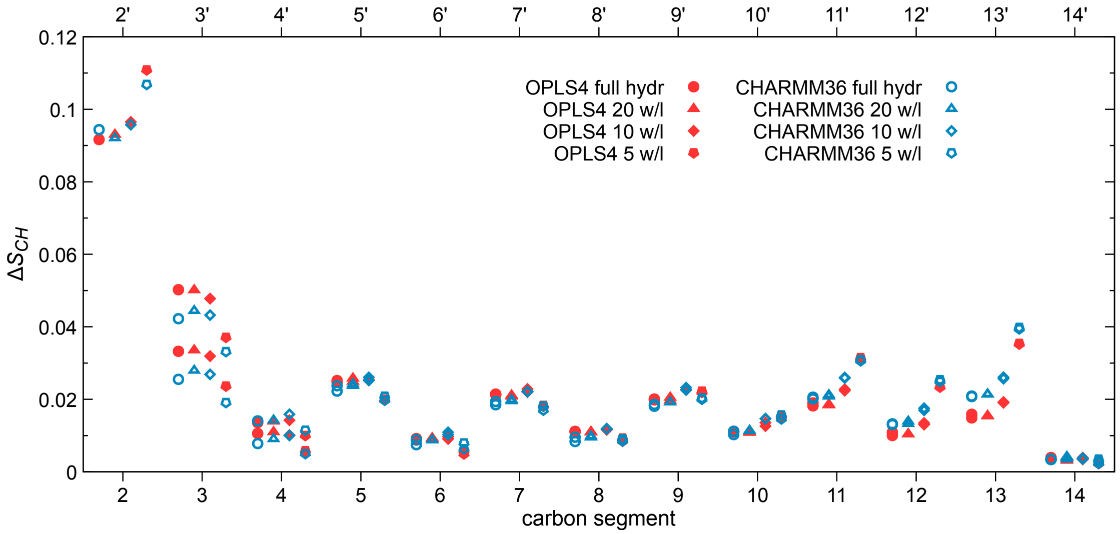

Figure S5: Difference in C–H bond order parameters between the corresponding carbon segments of the two DMPC lipid tails, $\left|S^{i'}_{\mathrm{CH}} - S^{i}_{\mathrm{CH}}\right|$, highlighting the odd/even variation visible in the main text Fig. 3A.

4 Complete plots of hydrocarbon tail order parameters upon dehydration

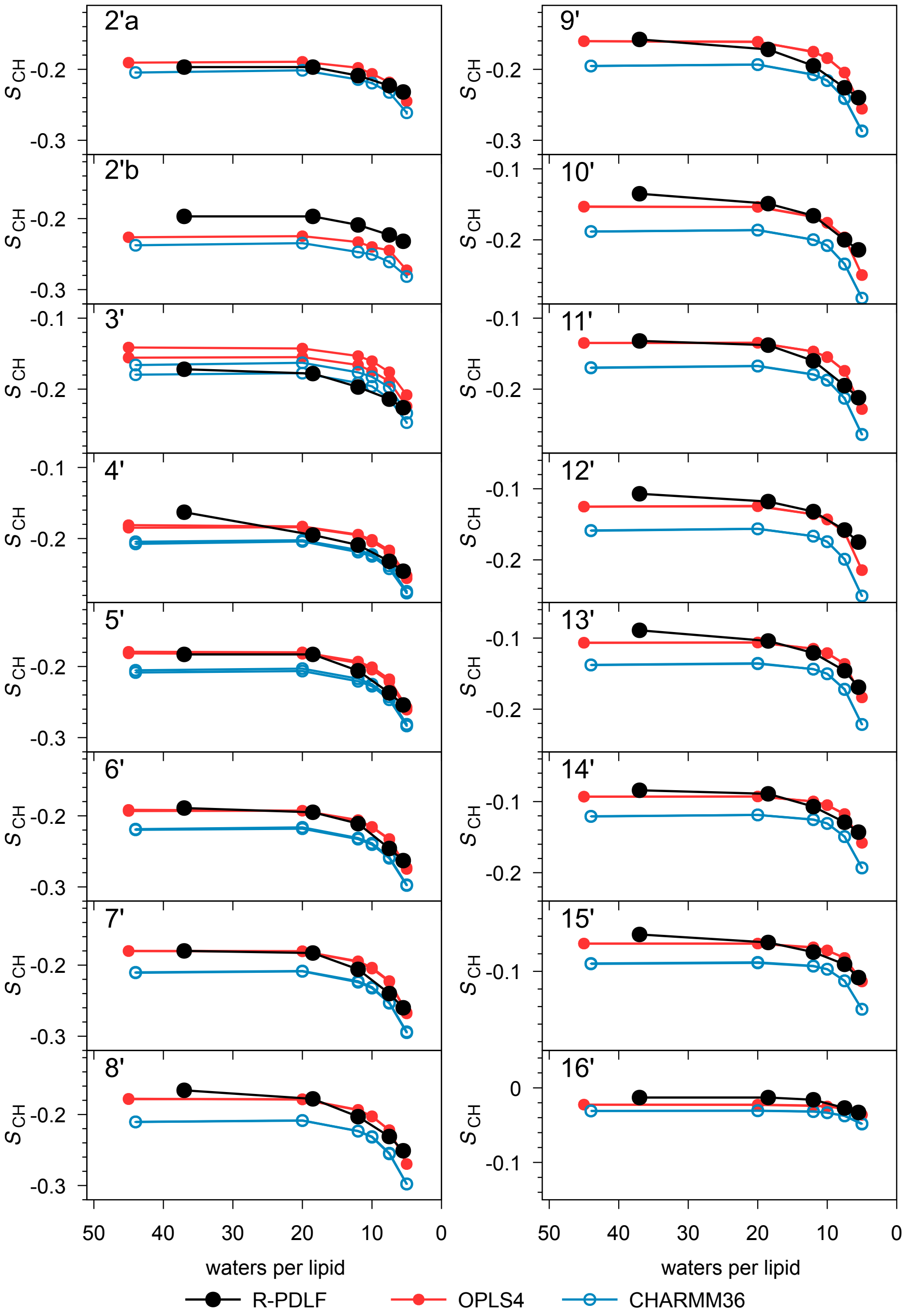

Figure S6: **Order parameters for palmitoyl tail of POPC as a function of lowering hydration** in OPLS4 and CHARMM36 force fields.

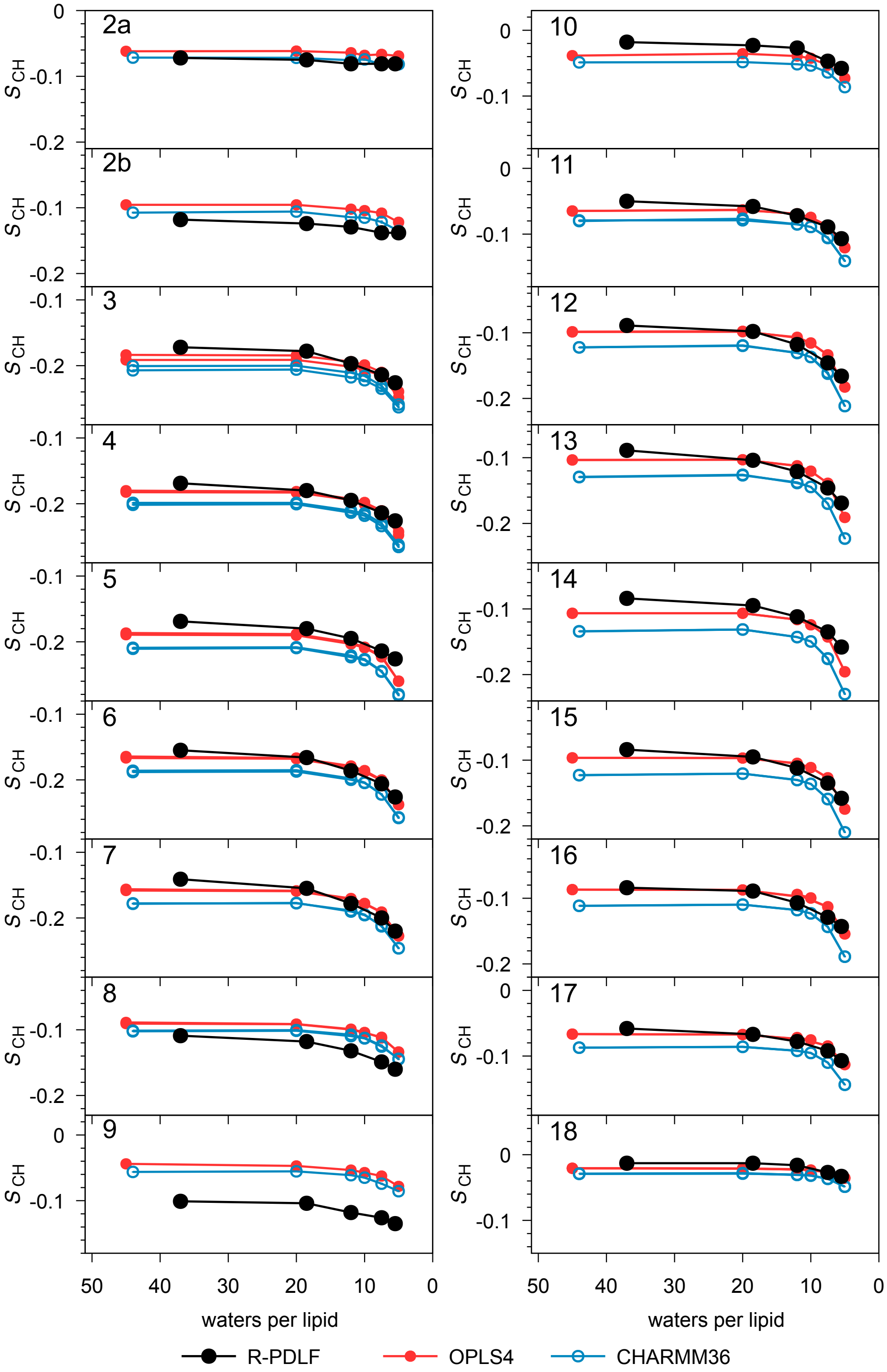

Figure S7: **Order parameters for oleoyl tail of POPC as a function of lowering hydration** in OPLS4 and CHARMM36 force fields.

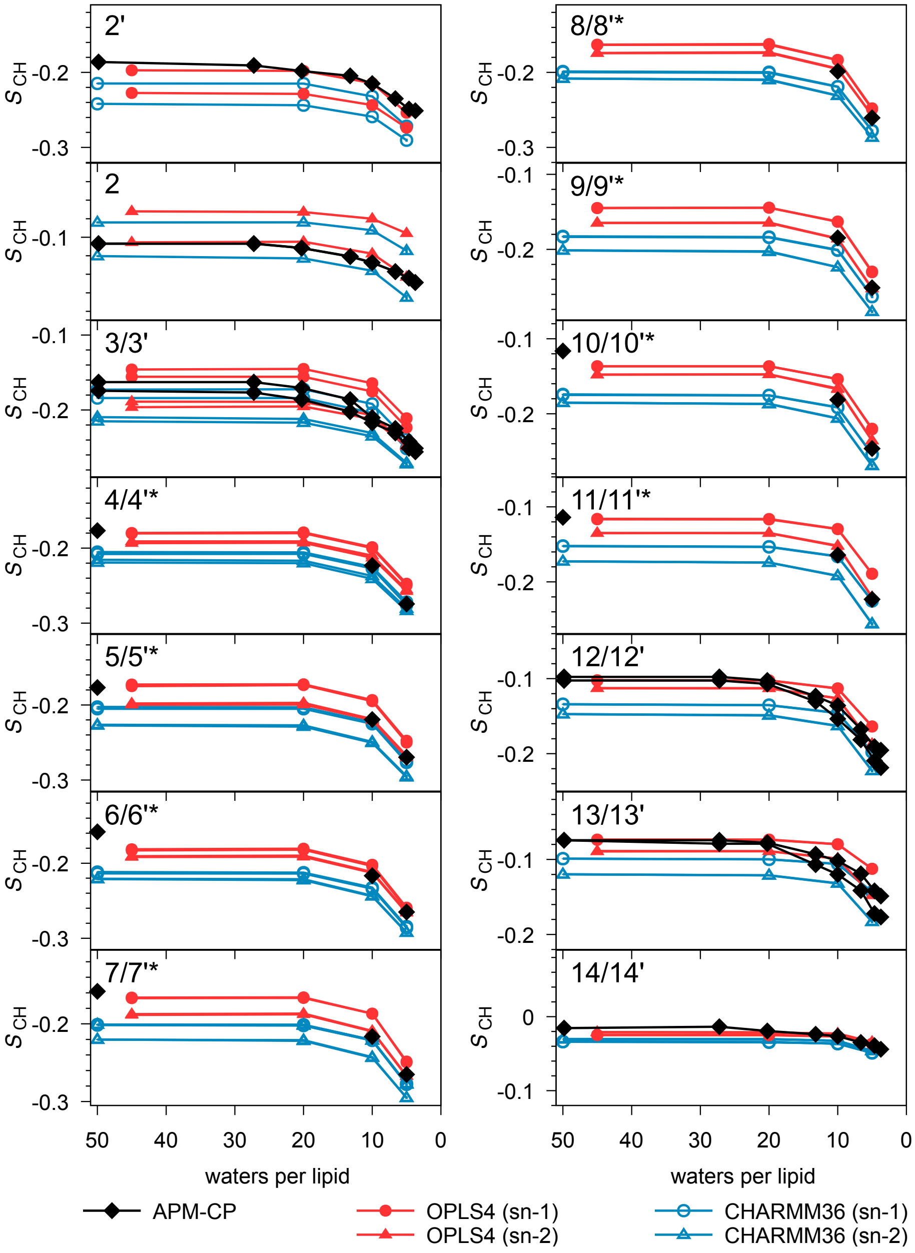

Figure S8: **Order parameters for the tails of DMPC as a function of lowering hydration** in OPLS4 and CHARMM36 force fields. Experimental data are from Ref 1. Carbons within the crowded region in the NMR spectra are marked with a star (*).

5 Tail ordering in MD

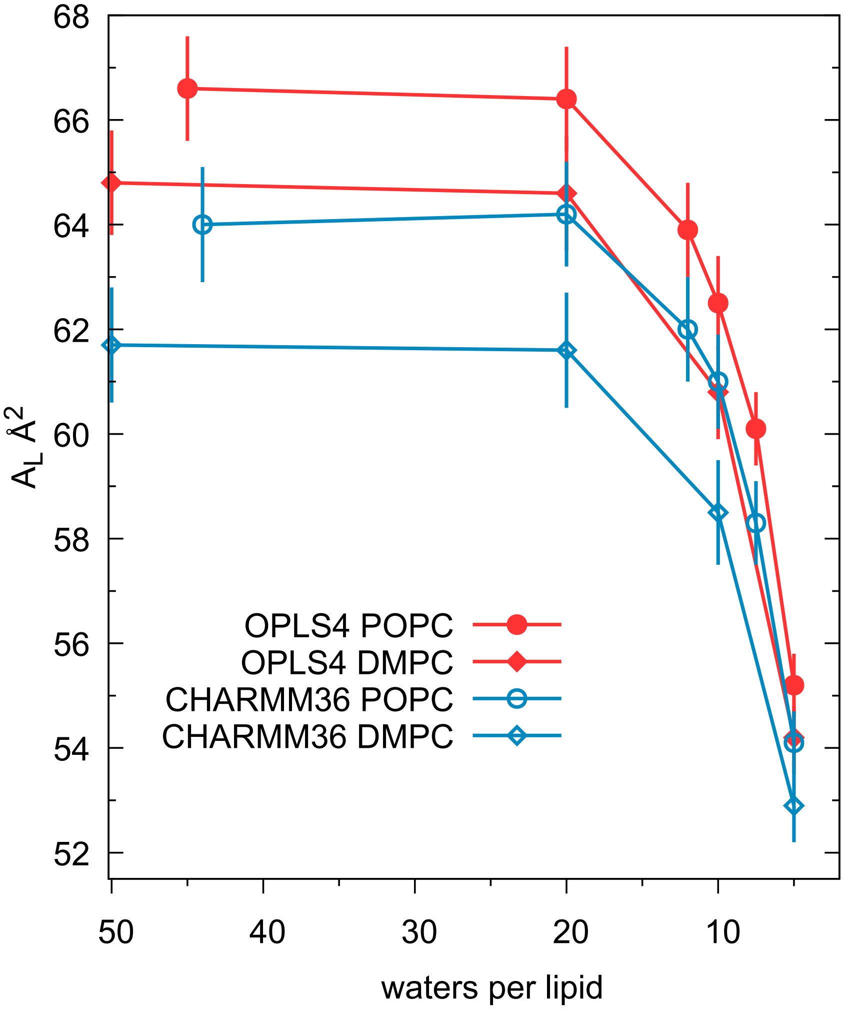

Figure S9: **Area per lipid in simulations as a function of hydration level.** Table S1 contains numerical values of the here plotted data.

Table S1: Equilibrium areas per lipid for POPC and DMPC at decreasing hydration.*

	OPLS4		CHARMM36	
	POPC	DMPC	POPC	DMPC
Full hydration	66.6 ± 1.0	64.8 ± 1.0	64.0 ± 1.1	61.7 ± 1.1
20 w/l	66.4 ± 1.0	64.6 ± 1.1	64.2 ± 1.0	61.6 ± 1.1
12 w/l	63.9 ± 0.9	—	62.0 ± 1.0	—
10 w/l	62.5 ± 0.9	60.8 ± 0.9	61.0 ± 0.9	58.5 ± 1.0
7.5 w/l	60.1 ± 0.7	—	58.3 ± 0.8	—
5 w/l	55.2 ± 0.6	54.2 ± 0.6	54.1 ± 0.6	52.9 ± 0.7

* Both mean and error (standard deviation) in Å^2.

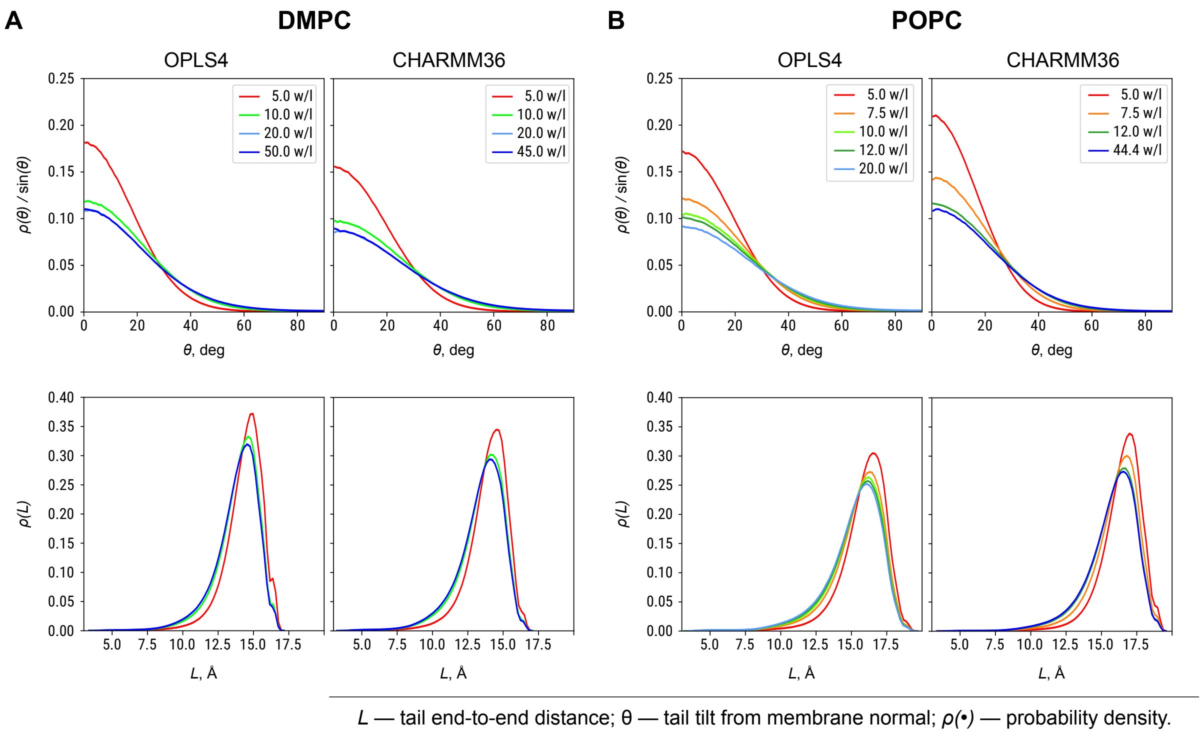

Figure S10: **Saturated *sn*-1 tail geometrical properties of DMPC (A) and POPC (B) lipids from MD simulations at different hydration states**. Tail tilt θ is an angle between end-to-end vector and bilayer normal. Its probability density distribution is normalized by the uniform-angle probability density distribution ($\times\frac{1}{\sin(\theta)}$).

6 Comparison of OPLS3e and OPLS4 force fields

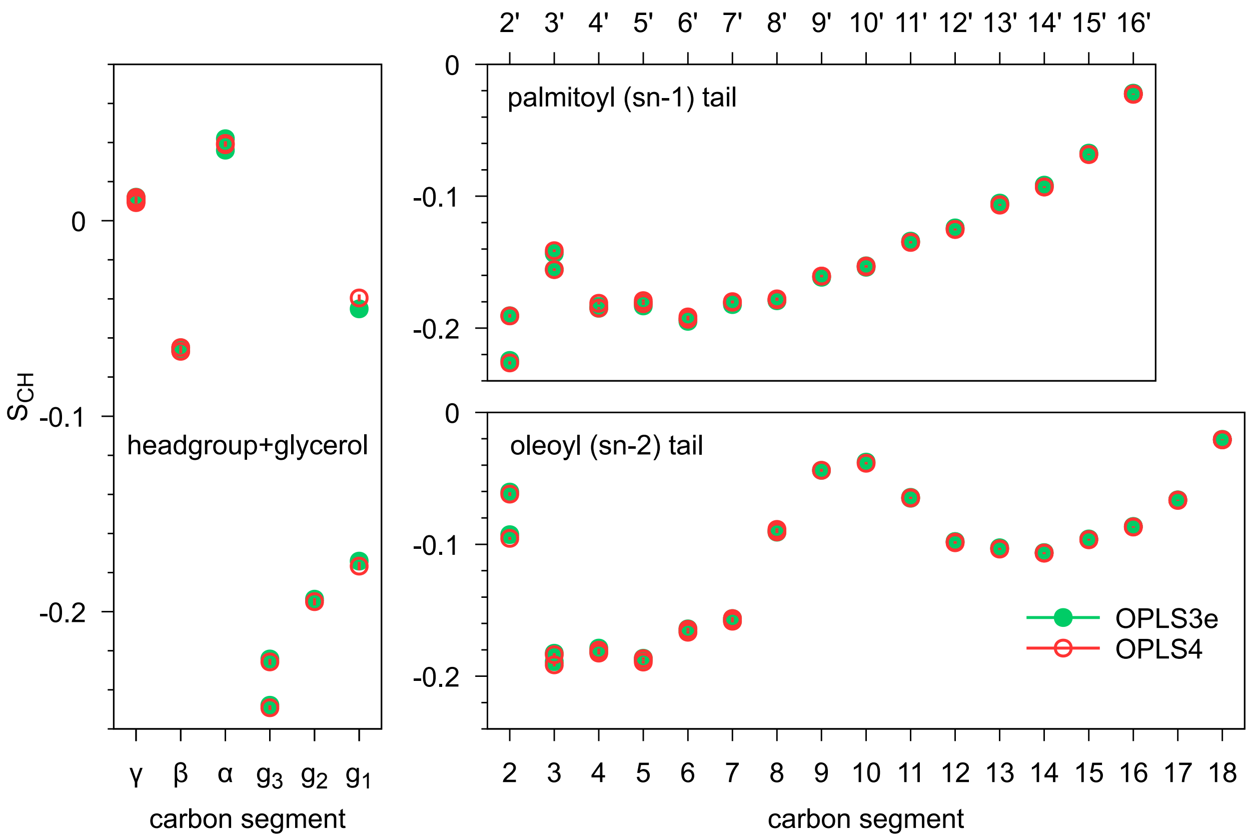

Figure S11: **POPC lipid C–H bond order parameters in OPLS4 and OPLS3e force fields at full hydration** for headgroup, backbone, and acyl chains compared to experiments. OPLS3e results are from Ref. 2.

At full hydration, C–H bond order parameters S_{CH} for OPLS4 seem to follow closely previously acquired OPLS3e data in headgroup, glycerol and tail segments, and the accuracy seems indistinguishable between these force fields at full hydration for POPC (Fig. S11).

Effect of dehydration in OPLS4 and OPLS3e for POPC membrane. At low water level (5 waters/lipid), there seems to be variation between OPLS4 and OPLS3e in C–H bond order parameters S_{CH} within head and glycerol group, but within tail segments force fields seem to be similar (Figs. S12, S13). The α carbon exhibits additional forking in OPLS4 (as in CHARMM36, see Fig. 4), which is not appearing in OPLS3e or at higher hydration

states. Also, order parameters of g_3, g_2 and g_{1R} carbons are diverging between OPLS4 and OPLS3e.

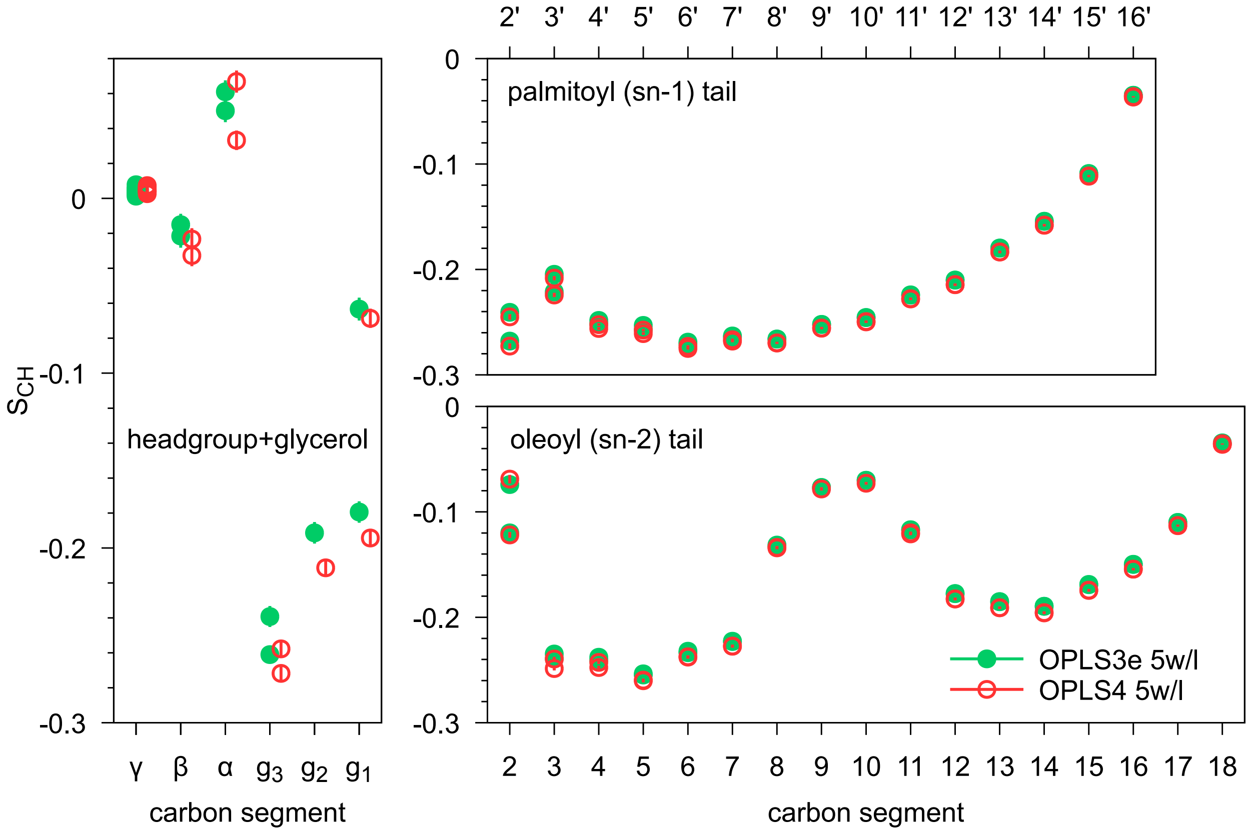

Figure S12: POPC lipid C–H bond order parameters in OPLS4 and OPLS3e force fields at low hydration (5 waters/lipid).

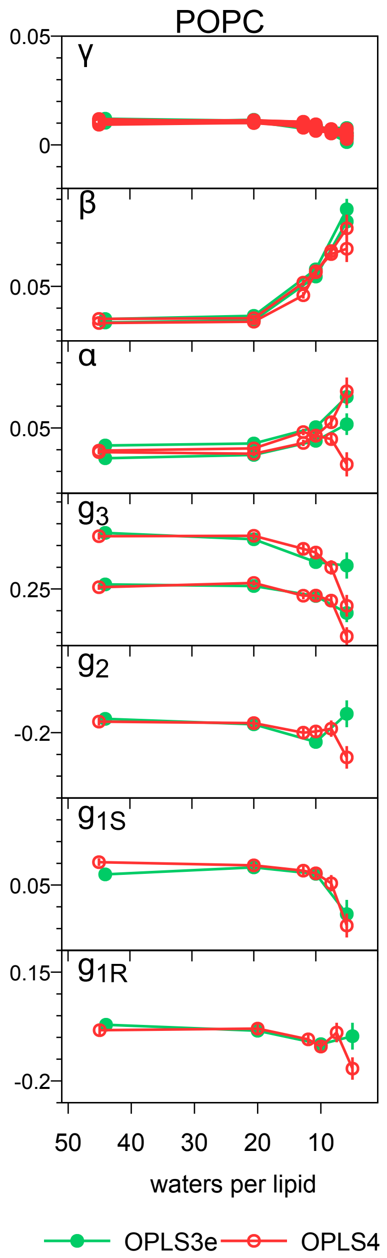

Figure S13: Order parameters as a function of lowering hydration in OPLS4 and OPLS3e for the head group of POPC.

Effect of salt in OPLS4 and OPLS3e for POPC membrane. We performed simulations with additional NaCl and $CaCl_2$ at 1000 mM concentration (simulation lengths 500 ns and 1 000 ns, respectively) to see whether there were changes in force field performance related to additional salt.

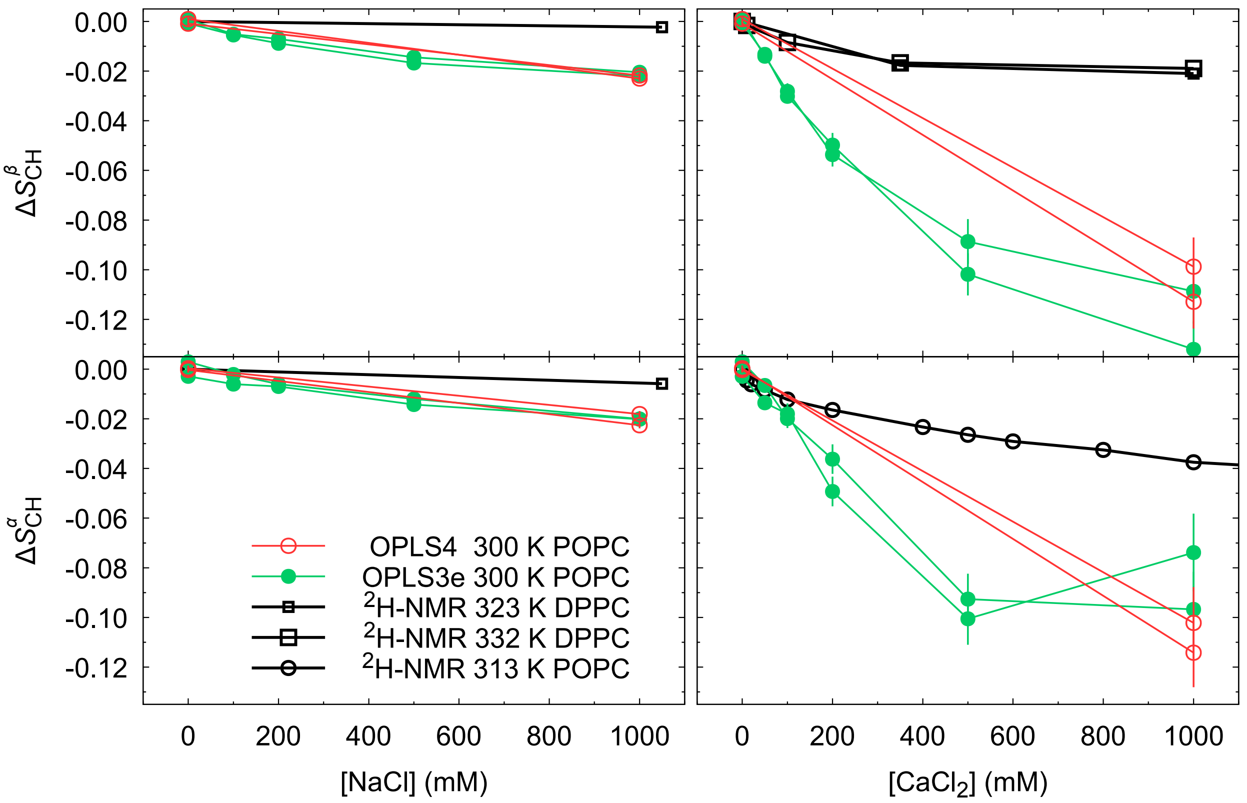

Figure S14: Change of order parameters in the headgroup α (lower panels) and β (upper panels) segments in response to of NaCl (left panels) or $CaCl_2$ (right panels) in the OPLS3e and OPLS4 force fields. Experimental values for DPPC (^{2}H NMR) at 323 K and 332 K are from Ref. 3 and for POPC (^{2}H NMR) at 313 K from Ref. 4. The average of the C–H bond order parameters of the R and S hydrogens was used to set the baseline. OPLS3e data are from Ref. 2.

Order parameter changes (Fig. S14) and ion distributions (Fig. S15) suggest that no improvement has been implemented in relation to ion–membrane interactions. As OPLS3e, also OPLS4 overestimates ion binding to the membrane, NaCl accumulation slightly and $CaCl_2$ accumulation dramatically. Therefore OPLS4, as most of the other currently available force fields, fails to provide correct description of cation binding to the membrane. OPLS4 should be used with caution if salt is added to the membrane system, especially calcium salt.

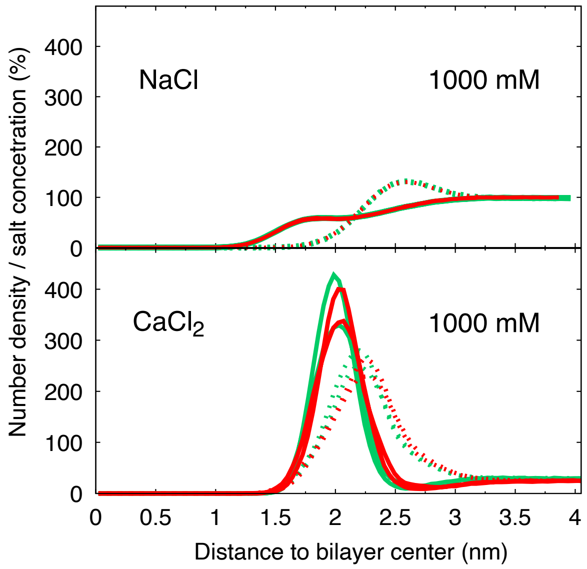

Figure S15: Distribution of ions in OPLS4 (red) and OPLS3e (green). Cations (Na^+ or Ca^{2+}) are shown as solid lines and Cl^- as dashed lines. Due to very slow equilibration of the $CaCl_2$ the last 100 ns of the 1 µs simulation was used. Note that both leaflets are plotted (two mostly overlapping lines).

References

(1) Dvinskikh, S. V.; Castro, V.; Sandström, D. Probing segmental order in lipid bilayers at variable hydration levels by amplitude- and phase-modulated cross-polarization NMR. *Phys Chem Chem Phys* **2005**, *7*, 3255–3257.

(2) Kurki, M.; Poso, A.; Bartos, P.; Miettinen, M. S. Structure of POPC Lipid Bilayers in OPLS3e Force Field. *J Chem Inf Model* **2022**, *62*, 6462–6474.

(3) Akutsu, H.; Seelig, J. Interaction of metal ions with phosphatidylcholine bilayer membranes. *Biochemistry* **1981**, *20*, 7366–7373.

(4) Altenbach, C.; Seelig, J. Calcium binding to phosphatidylcholine bilayers as studied by deuterium magnetic resonance. Evidence for the formation of a calcium complex with two phospholipid molecules. *Biochemistry* **1984**, *23*, 3913–3920.
